# Respiratory syncytial virus phosphoprotein has NTPase and helicase-like activities

**DOI:** 10.1128/jvi.00996-25

**Published:** 2025-09-25

**Authors:** Ting Shu, Xiaotong Wang, Yiyang Li, Jiaoling Su, Haiwu Zhou, Mengyu Hu, Puyu Yang, Chao Shan, Yang Qiu, Xi Zhou

**Affiliations:** 1 State Key Laboratory of Virology and Biosafety, Wuhan Institute of Virology, Chinese Academy of Sciences 74614 , Wuhan, China; 2 University of Chinese Academy of Sciences, Beijing, China; 3 State Key Laboratory of Virology and Biosafety, Hubei Provincial Research Center for Basic Biological Sciences, College of Life Sciences, Wuhan University 98436, Wuhan, China; Loyola University Chicago - Health Sciences Campus, Maywood, Illinois, USA

**Keywords:** helicase-like activity, respiratory syncytial virus, phosphoprotein, NTPase acitivity

## Abstract

Respiratory syncytial virus (RSV), a non-segmented, negative-sense RNA virus (NNSV) in the family Pneumoviridae, represents a significant global health burden causing severe lower respiratory tract infections in infants and immunocompromised adults. While RNA helicases are essential for viral replication through their RNA remodeling functions, the presence of such enzymatic activities in RSV remains unclear. Here, we reveal that the RSV phosphoprotein (P), despite lacking canonical helicase motifs, demonstrates nucleoside triphosphatase (NTPase) activity and directional 5′-to-3′ RNA helix-unwinding capacity in an NTP-dependent manner. Through mutagenesis assays, we establish a functional coupling between NTP hydrolysis and helicase-like activity. Importantly, reverse genetics experiments, RSV minigenome, and antiviral-effect assays demonstrate the essentiality of RSV P’s helicase-like activity for viral viability and replication. These findings identify P as an enzymatic component critical for RSV replication, providing new insights into the mechanisms of pneumovirus propagation.

**IMPORTANCE:**

RNA helicases and helicase-like viral proteins are crucial for viral RNA replication and are prime targets for antiviral development. RSV infects nearly all children by age two, causing over 30 million acute lower respiratory infections, 3.6 million hospitalizations, and 100,000 deaths annually in children under five, while also posing a significant threat to immunocompromised adults and the elderly. In this study, we demonstrate for the first time that the RSV P has NTPase activity and unwinds RNA helices in an NTP-dependent manner. Mutagenesis and reverse genetics experiments confirm that these enzymatic activities are essential for RSV viability. These findings not only redefine RSV P as a multifunctional protein but also expand our understanding of the RSV replication machinery, highlighting the potential of targeting P for antiviral therapy.

## INTRODUCTION

Respiratory syncytial virus (RSV), a non-segmented negative-sense single-stranded RNA virus (NNSV) of the *Orthopneumovirus* genus (family *Pneumoviridae*) (1), represents a significant global health burden. As a ubiquitous pathogen, RSV infects nearly all children by the age of two and is responsible for over 30 million acute lower respiratory tract infections annually in children under five, leading to 3.6 million hospitalizations and approximately 100,000 deaths worldwide. Beyond pediatric populations, RSV causes substantial morbidity and mortality in immunocompromised adults and the elderly, underscoring its serious threat to vulnerable age groups (2). Despite decades of research, therapeutic and prophylactic options remain limited, necessitating deeper insights into the molecular mechanisms governing RSV replication and pathogenesis.

The RSV genome spans approximately 15.2 kilobases (kb) and encodes 11 proteins through 10 genes (1). Among these, the nucleoprotein (N), phosphoprotein (P), and large polymerase protein (L) form the minimal replication complex essential for viral propagation. The N protein encapsidates viral RNAs to form a ribonucleoprotein (RNP) complex, shielding it from host innate immune sensors and nucleases. In contrast, P protein serves as a critical cofactor for the L polymerase, anchoring it to the N-RNA template while preventing premature binding of nascent N to host RNAs (3–5).

RNA helicases, a class of enzymes conserved across diverse RNA viruses, are indispensable for viral RNA replication and transcription. These enzymes utilize nucleoside triphosphate (NTP) hydrolysis to unwind structured RNA duplexes, resolve secondary structures, and separate genomic strands during replication (6–8). Currently, a wide range of RNA viruses have been reported to encode viral proteins with RNA helicase or helicase-like activities, including picornavirus 2C (9), enterovirus 71 2C (10), norovirus NS3 (11), flavivirus NS3 (12, 13), alphavirus nsP2 (14), coronavirus Nsp13 (15, 16), cypovirus VP5 (17), and filovirus VP35 (18). These RNA helicase or helicase-like viral proteins play pivotal roles in the viral RNA replication and therefore are recognized to be ideal targets for developing antivirals (19–22). However, since bioinformatic analyses of RSV-encoded proteins reveal no conserved canonical NTPase/helicase motifs, it is still unknown whether RSV or any member in the family *Pneumoviridae* encodes proteins with RNA helicase or helicase-like activity, representing a critical gap in understanding RSV replication strategies and underscoring the need to re-evaluate the functional repertoire of RSV proteins.

Our previous study reported that VP35 encoded by Ebola virus (EBOV), a high-risk pathogenic virus belonging to NNSV, contains RNA helicase-like activity that can hydrolyze all types of NTPs and unwind RNA helices in an NTP-dependent manner (18). Interestingly, EBOV VP35 also has no conserved canonical NTPase/helicase motifs. Although EBOV VP35 is different from other NNSV-encoded P proteins, they are considered functional analogs in NNSVs due to conserved functional modules and roles in viral replication (20, 21). Therefore, it would be intriguing to exploit whether RSV P also has a helicase-like activity similar to that of EBOV VP35.

In the current study, we demonstrate, for the first time, that RSV P exhibits intrinsic NTPase activity and unwinds RNA helices in a 5′-to-3′ direction, dependent on NTP hydrolysis. Crucially, mutagenesis experiments confirm that the NTPase function is indispensable for the helicase-like activity of RSV P, while reverse genetics, RSV minigenome, and antiviral-effect assays reveal that disrupting these enzymatic properties severely compromises RSV viability. These findings not only redefine RSV P as a multifunctional protein but also extend existing paradigms of RSV replication machinery.

## RESULTS

### RSV P has NTPase activity

**Fig 1:**
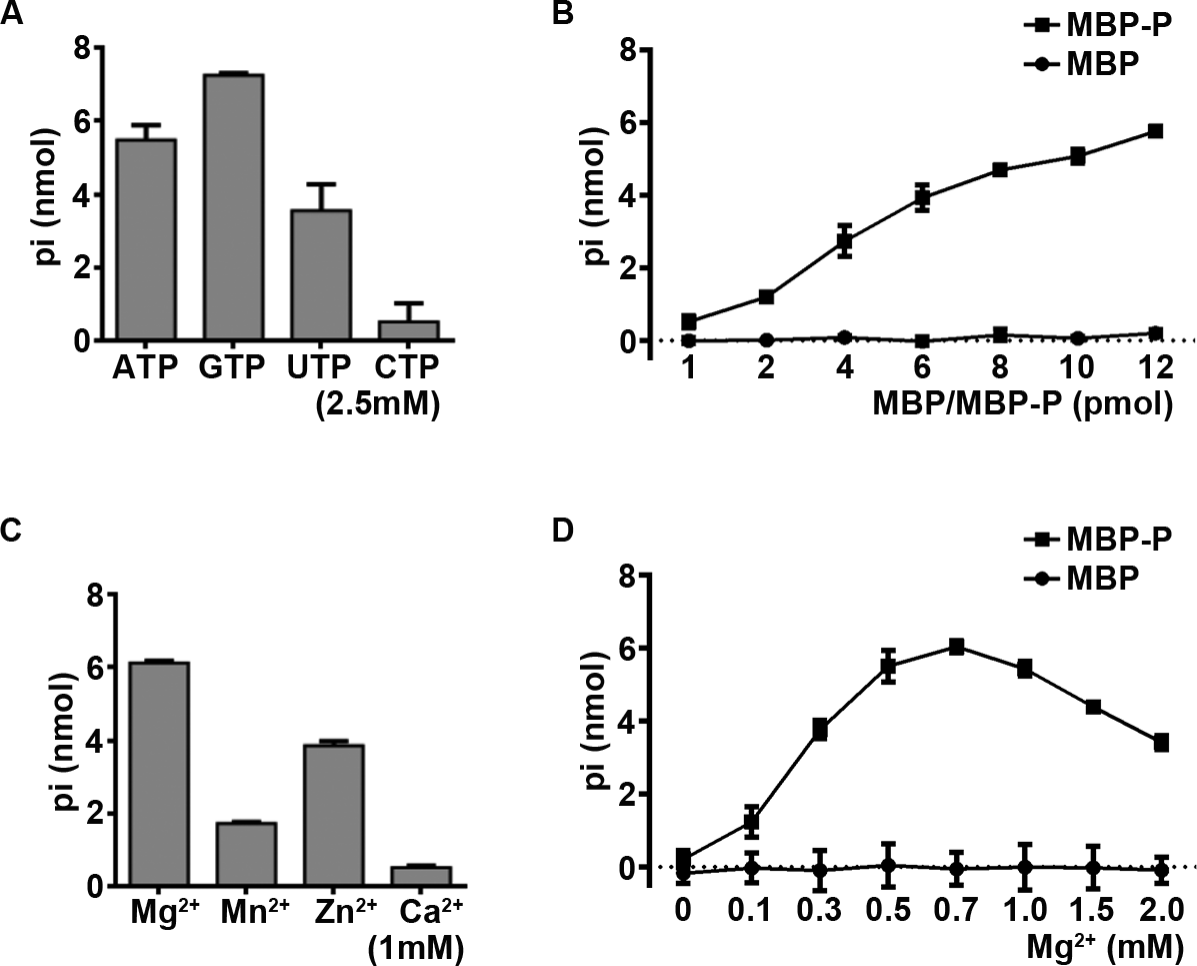
RSV P has NTPase activities. (**A**) 20 pmol MBP-P was incubated with the indicated NTPs (2.5 mM). The NTPase activity was measured as nanomoles of released inorganic phosphate (Pi) by using a sensitive colorimetric assay. (**B**) 2.5 mM ATP was incubated with MBP or MBP-P at the increasing concentrations, respectively. (**C**) 20 pmol MBP-P was reacted with 2.5 mM ATP and 1 mM of the indicated divalent metal ions. (**D**) 20 pmol MBP or MBP-P was reacted with 2.5 mM ATP at the indicated concentrations of MgCl2. Error bars represent standard deviation (SD) values from three separate experiments.

To investigate whether RSV P has RNA helicase-like activity, we first examined its NTPase activity, as the ability to hydrolyze NTPs is a common feature of conserved viral RNA helicases. To this end, we expressed and purified RSV P fused with maltose-binding protein (MBP) at its N-terminal (MBP-P) using a baculovirus expression system (Fig. S1) and tested the NTPase activity of MBP-P via using a canonical colorimetric assay. In this assay, the activity of hydrolyzing NTPs (ATP, GTP, UTP, and CTP) was measured by the total amounts of free orthophosphate released after NTP was hydrolyzed. As shown in Fig. 1A, we found that RSV P can efficiently hydrolyze ATP, GTP, and UTP, with a preference for GTP. Since ATP is the common hydrolytic substrates in colorimetric assay, we used it in the subsequent assays. We showed that MBP-P can hydrolyze ATP in a dose-dependent manner (Fig. 1B). Moreover, we then tested the optimal biochemical reaction conditions for RSV P, including different salt content and Mg^2+^ concentrations. We found that 1 mM Mg^2+^ or Zn^2+^ can support the ATPase activity of MBP-P (Fig. 1C). And the ATPase activity of MBP-P was dependent on divalent metal ions, as its ATP-hydrolyzing activity was undetectable in the absence of Mg^2+^ (0 mM). MBP-P reached the highest ATPase activity at a concentration of 0.7 mM Mg^2+^, while higher concentrations of Mg^2+^ showed certain inhibitory effects on the ATPase activity (Fig. 1D).

Taken together, our data indicated that RSV P has NTPase activity that is dependent on the presence of certain divalent metallic ions.

### RSV P contains dsRNA-binding activity and RNA helix-unwinding activity

**Fig 2:**
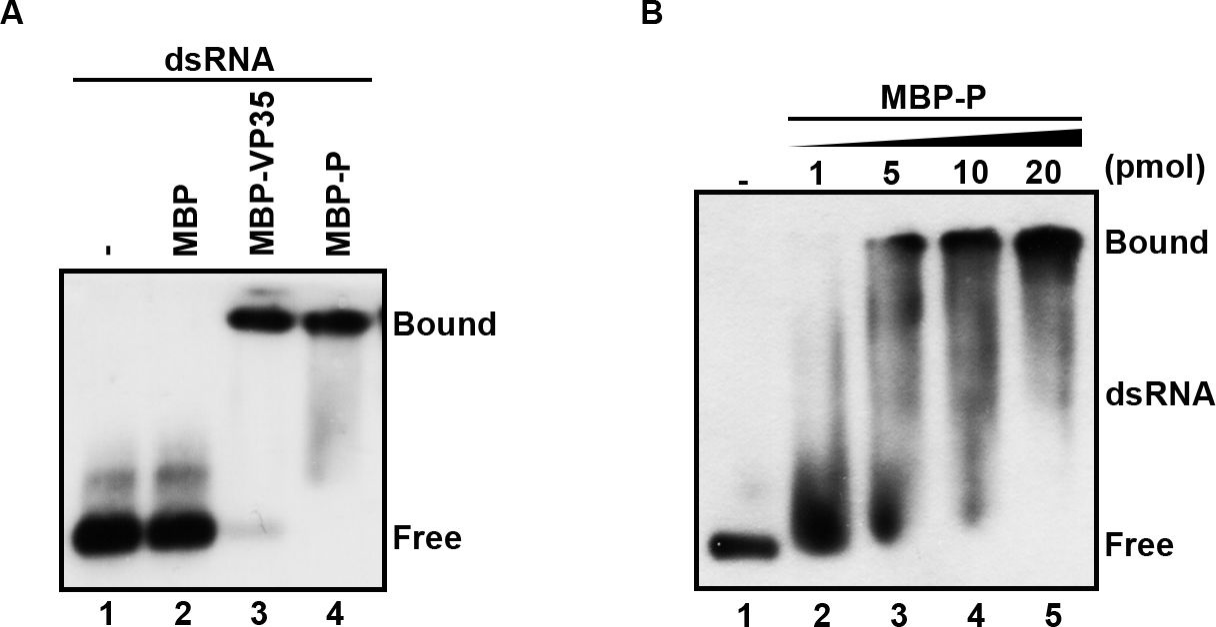
RSV P has dsRNA-binding activity. (**A**) Gel mobility shift assays were performed to evaluate the dsRNA-binding activity of MBP-P. Protein was incubated with 0.1 pmol DIG-labeled dsRNA substrate, and the complex was analyzed by gel electrophoresis, transferred to membranes, and then incubated with anti-DIG antibody conjugated with alkaline phosphatase. Lane 1: no protein supplemented. Lane 2: 20 pmol MBP supplemented. Lane 3: 20 pmol MBP-VP35 supplemented. Lane 4: 20 pmol MBP-P supplemented. (**B**) Gel mobility shift assays were performed by incubating the indicated increasing concentrations (1–20 pmol) of MBP-P with 0.1 pmol dsRNA.

After identifying that RSV P has NTPase activity, we then examined whether it possesses dsRNA-binding activity via incubating MBP-P with digoxin (DIG)-labeled dsRNA derived from 1- to 200-nt *egfp* ORF. Our results showed that MBP-P can bind to dsRNA in a dose-dependent manner, while the negative control (MBP alone) does not exhibit dsRNA-binding activity (Fig. 2A and B).

**Fig 3:**
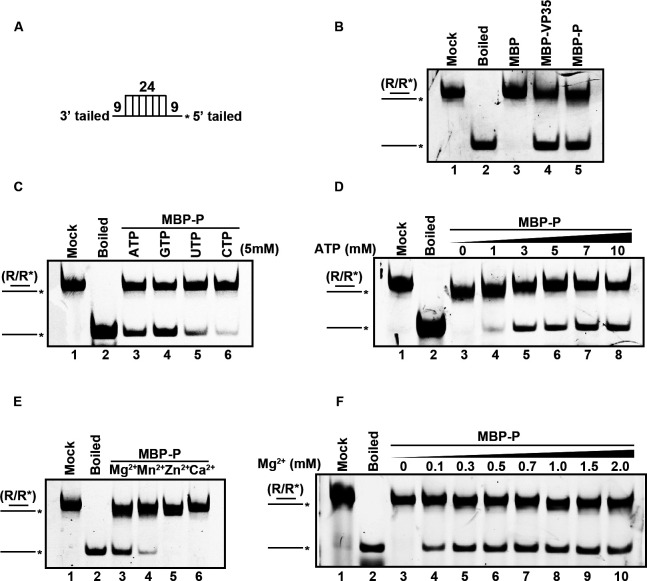
RSV P has helix-unwinding activity. (**A**) Schematic illustration of the standard RNA helix substrate (R/R*). Asterisks indicate the HEX-labeled strands. (**B**) The standard RNA helix substrate (0.1 pmol) was incubated with 20 pmol of MBP, MBP-VP35, and MBP-P (lanes 3–5). The unwinding activity was assessed via gel electrophoresis and scanning on a Typhoon 9500 imager. Non-boiled reaction mixture (lane 1) and reaction mixture with MBP (lane 3) were used as negative controls, and boiled reaction mixture (lane 2) and reaction mixture with MBP-VP35 (lane 4) were used as positive controls. (**C**) The RNA helix-unwinding assay was performed in the presence of each indicated NTPs (5 mM). (**D**) The RNA helix-unwinding assay was performed in the presence of increasing concentrations of ATP. (**E**) 20 pmol of MBP-P was incubated with 0.1 pmol standard RNA helix substrate at 1 mM indicated divalent metal ions. (**F**) 20 pmol MBP-P was incubated with 0.1 pmol standard RNA helix substrate in the presence of increasing concentrations of MgCl2.

Because RSV P possesses NTPase and dsRNA-binding activity, we then examined whether it has RNA helix-unwinding activity using a classical helix-unwinding assay as described previously (18). In this assay, a hexachlorofluorescein (HEX)-labeled RNA helix substrate with both 5′ and 3′ single-strand protrusions was constructed (Fig. 3A; Table S2) and subjected to incubation with MBP-P in the standard unwinding reaction containing ATP and Mg^2+^ and then separated the substrate via gel electrophoresis. As shown in Fig. 3B, the HEX-labeled RNA strand was efficiently released from the RNA helix substrate in the presence of MBP-P (lane 5), while the same substrate remained stable when MBP alone was added in the reaction as negative control (lane 3). The boiled reaction mixture (lane 2) and the addition of MBP-fusion EBOV VP35 (MBP-VP35, lane 4), a well-characterized viral RNA helicase-like protein, were used as positive controls.

We further examined the helix-unwinding activity of MBP-P under four types of NTPs. The results showed that MBP-P can efficiently unwind the RNA helix substrate in the presence of ATP and GTP, whereas its RNA helix-unwinding efficiency was relatively weak under UTP and CTP (Fig. 3C). Moreover, we found that the helix-unwinding activity of MBP-P can be stimulated by ATP in a dose-dependent manner (Fig. 3D).

We further characterized the roles of divalent metallic ions in RNA helix-unwinding of RSV P. From the results, we found that Mg^2+^ and Mn^2+^ can support MBP-P in unwinding RNA helix substrate, especially Mg^2+^ (Fig. 3E). In addition, the RNA helix unwinding of RSV P required the presence of divalent metallic ions, as no RNA strand can be released by MBP-P in the absence of Mg ^2+^(Fig. 3F, lane 3). Moreover, MBP-P exhibited optimal helix-unwinding activity in the presence of 0.7 mM Mg^2+^, consistent with the previous results (Fig. 1D).

Taken together, our results demonstrated that RSV P has dsRNA-binding activity and RNA helix-unwinding activity. The RNA helix-unwinding activity of RSV P is dependent on ATP and divalent metal ions.

### Characterization of the RNA helix-unwinding activity of RSV P

**Fig 4:**
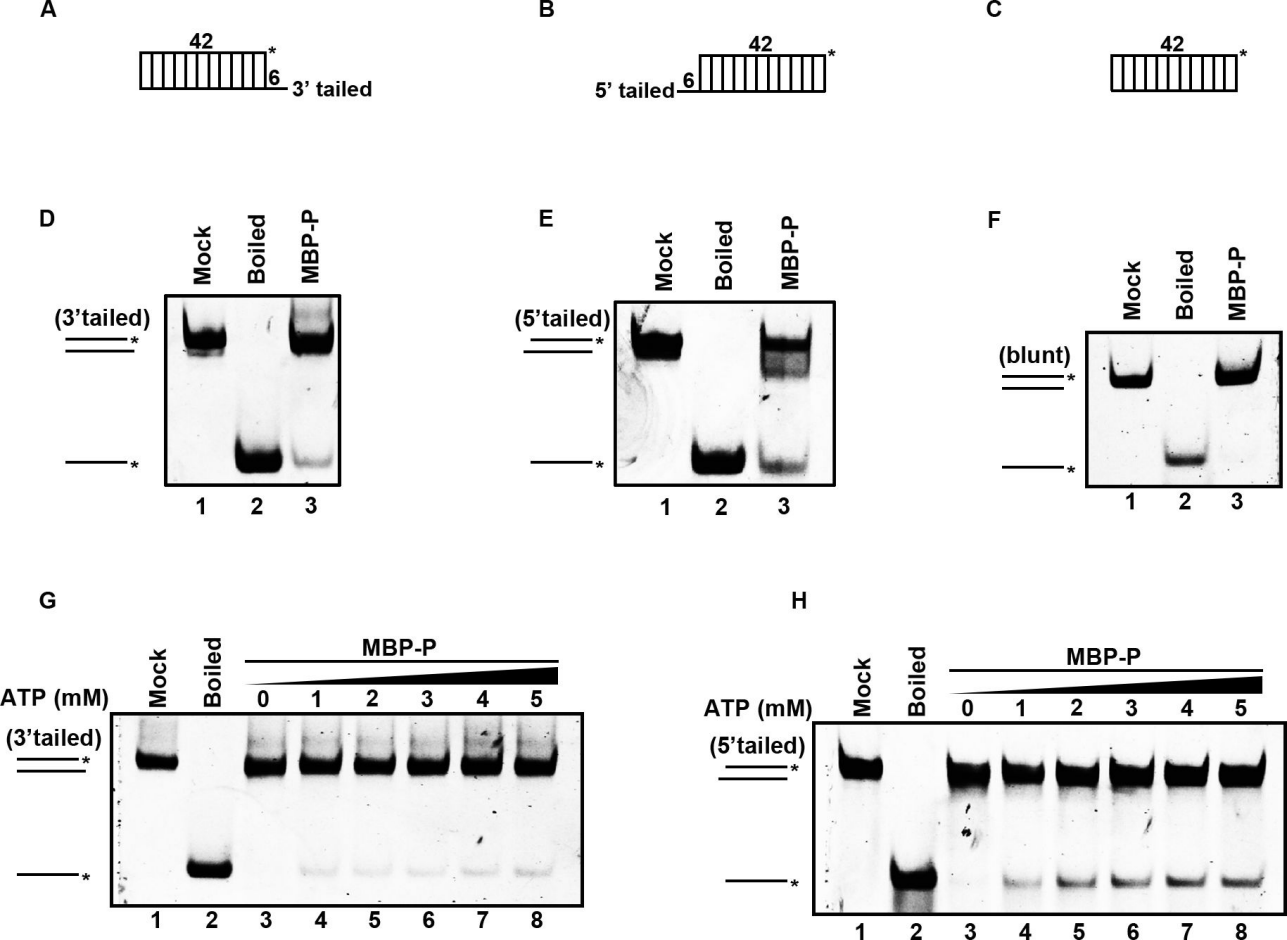
RSV P unwinds RNA helix in the 5′ to 3′ directionality. (**A–C**) Schematic illustrations of the RNA helix substrates with 3′ tailed (**A**), 5′ tailed (**B**), and blunt ends (**C**). Asterisks indicate the HEX-labeled strand. (**D–F**) 20 pmol MBP-P was reacted with 0.1 pmol 3′-tailed (**D**) or 0.1 pmol 5′-tailed (**E**) RNA helix substrate, or 0.1 pmol RNA helix substrate with blunt ends (**F**). (**G and H**) 20 pmol MBP-P was reacted with 0.1 pmol 3′-tailed (**G**) or 0.1 pmol 5′-tailed (**H**) RNA helix substrate in the presence of increasing concentrations of ATP.

After identifying that RSV P has RNA helix-unwinding activity, we then sought to determine its helix-unwinding directionality, as the directionality of helix-unwinding is a fundamental characteristic for helicases (16, 18). We generated three different RNA helix substrates: one containing a 3′ single-strand protrusion (Fig. 4A), one containing a 5′ single-strand protrusion (Fig. 4B), and the last one with blunt ends (Fig. 4C). Then, MBP-P was reacted with these three different RNA helices using the standard helix-unwinding assay. Interestingly, our data showed that MBP-P could unwind both 3′-tailed and 5′-tailed RNA helix (Fig. 4D and E), but not the blunt-ended one (Fig. 4F). Furthermore, MBP-P unwound 5′-tailed RNA helix more efficiently than the 3′-tailed one (Fig. 4E lane 3 and 4D lane 3). Moreover, the increasing concentrations of ATP failed to promote the unwinding rate of 3′-tailed RNA helix (Fig. 4G), but efficiently promoted the unwinding activity of RSV P with 5′-tailed RNA helix in a dose-dependent manner (Fig. 4H). Together, our findings showed that the RNA helix-unwinding activity of RSV P tends to be in the 5′-to-3′ direction.

**Fig 5:**
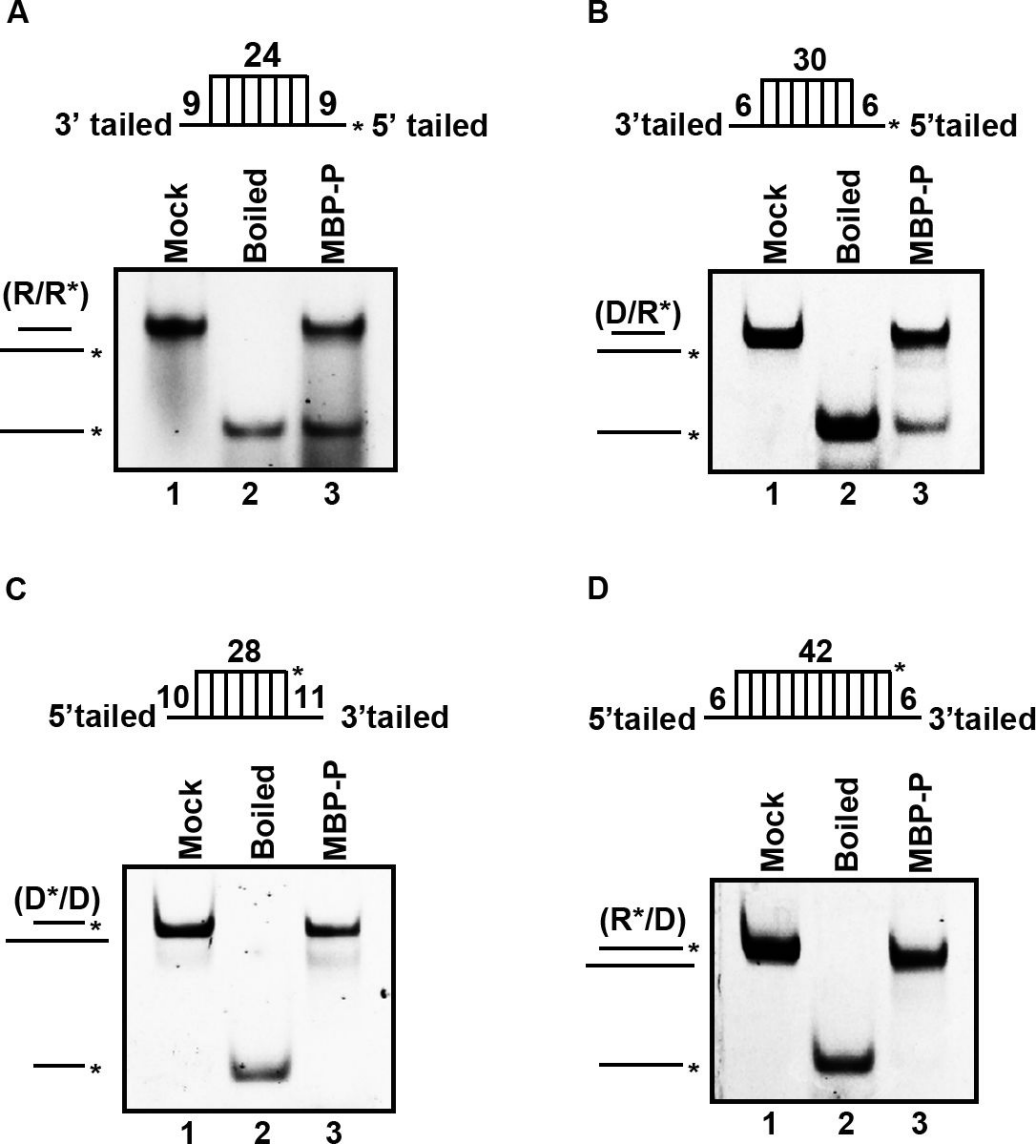
RSV P unwinds RNA-protruded nucleic acid helix. (**A–D**) 20 pmol MBP-P was reacted with 0.1 pmol of standard RNA helix (R/R*) (**A**), DNA/RNA hybrid with longer RNA strand (D/R*) (**B**), or DNA helix (D*/D) (**C**) or longer DNA strand (R*/D) (**D**). The schematic illustrations of the helix substrates are illustrated in the upper. Asterisks indicate the HEX-labeled strand.

We sought to examine whether RSV P can also unwind DNA helices or RNA-DNA hybrids. To this end, we constructed four different nucleic acid helix substrates: RNA helix (R*/R) with RNA strand protrusions, DNA helix (D*/D) with DNA strand protrusions, and RNA-DNA hybrids (D/R* or R*/D) with RNA or DNA strand protrusions (Fig. 5A through D, upper panel). Our results showed that MBP-P could unwind both R*/R (Fig. 5A) and D/R* (Fig. 5B), which have longer RNA strand protrusions. On the other hand, MBP-P could not unwind substrates with longer DNA strands, including D*/D (Fig. 5C) and R*/D (Fig. 5D).

Together, our findings indicate that RSV P requires the presence of protruded single-stranded RNA to unwind nucleic acids from 5′-to-3′ direction.

### The NTPase activity is required for the helix unwinding of RSV P

**Fig 6:**
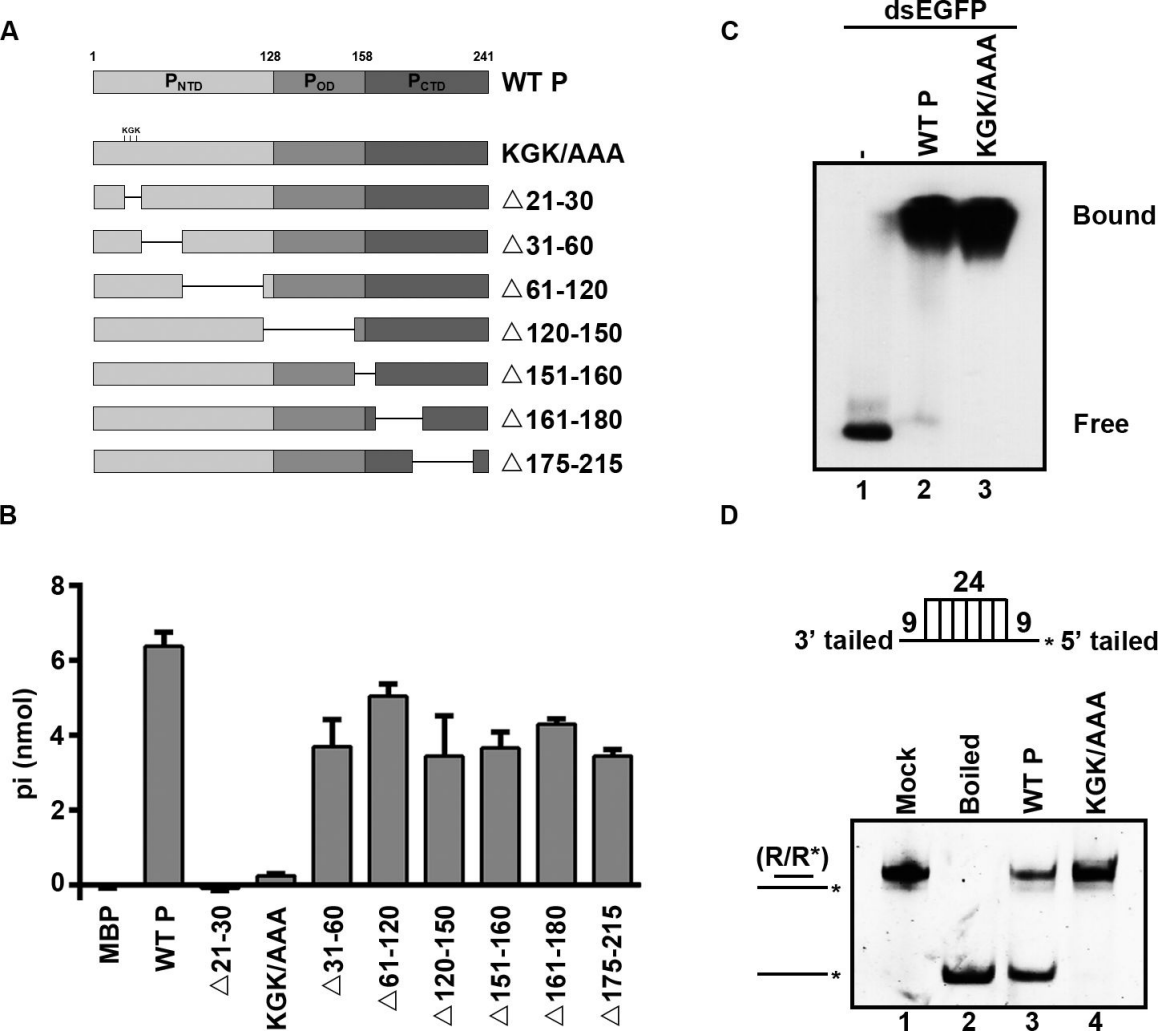
The NTPase activity of RSV P is required for its helix unwinding. (**A**) Schematic illustration of deletion mutations and point mutations. NTD is the N-terminal domain, OD is the oligomerization domain, and CTD is the C-terminal domain. The KGK sites are replaced with alanine. (**B**) 20 pmol of MBP-fusion WT P, △21–30, KGK/AAA, △31–60, △61–120, △120–150, △151–160, △161–180, and △175–215 were reacted with 2.5 mM ATP, respectively. The NTPase activity was measured as nanomoles of released inorganic phosphate (Pi) by using a sensitive colorimetric assay. (**C**) 0.1 pmol DIG-labeled dsRNA substrate was incubated with 20 pmol of WT P (lane 2) and KGK/AAA (lane 3), respectively. The complex was analyzed by gel electrophoresis, transferred to membranes, and then incubated with anti-DIG antibody conjugated with alkaline phosphatase. (**D**) Upper : Schematic illustration of the standard RNA helix substrate (R/R*); asterisks indicate the HEX-labeled strands. Lower : The RNA helix-unwinding assays were performed by incubating 0.1 pmol standard helix substrate with 20 pmol of each indicated protein.

The interaction between P and N is essential for RSV replication (5). The N-terminal 1–20 amino acids (a.a.) of the P contain multiple residues critical for N-P interaction, and deletion of residues 220–241 at the C-terminus of P abolishes its binding to N (23, 24). We sought to determine the key regions responsible for RNA helix-unwinding activity of RSV P. Given that P–N binding is essential for viral replication, we deliberately avoided these regions and generated a series of deletion mutations (25) (as illustrated in Fig. 6A; Fig. S2A) and found that RSV P mutant with the deletion of amino acids 21–30 (△21–30) failed to hydrolyze ATP, indicating that this region is important for the ATPase activity of RSV P. The a.a. GK is the conserved NTPase active-site signature of the classical superfamily 3 (SF3) helicases (10, 11). Based on this result, we further generated the point mutant (KGK/AAA) containing three crucial sites within a.a. 21–30 (K25, G26, and K27) being mutated to alanine. We found that the KGK/AAA mutant also completely lost its ATPase activity like △21–30 (Fig. 6B). Furthermore, we examined the dsRNA-binding and helix-unwinding activities of the KGK/AAA mutant and found that, although the KGK/AAA mutant retained its dsRNA-binding activity (Fig. 6C), it completely lost the RNA helix-unwinding activity (Fig. 6D; Fig. S2B). Our results indicate that NTPase activity is required for the helix-unwinding activity of RSV P.

### The RNA helicase activity of P is critical for the viability and replication of RSV

**Fig 7:**
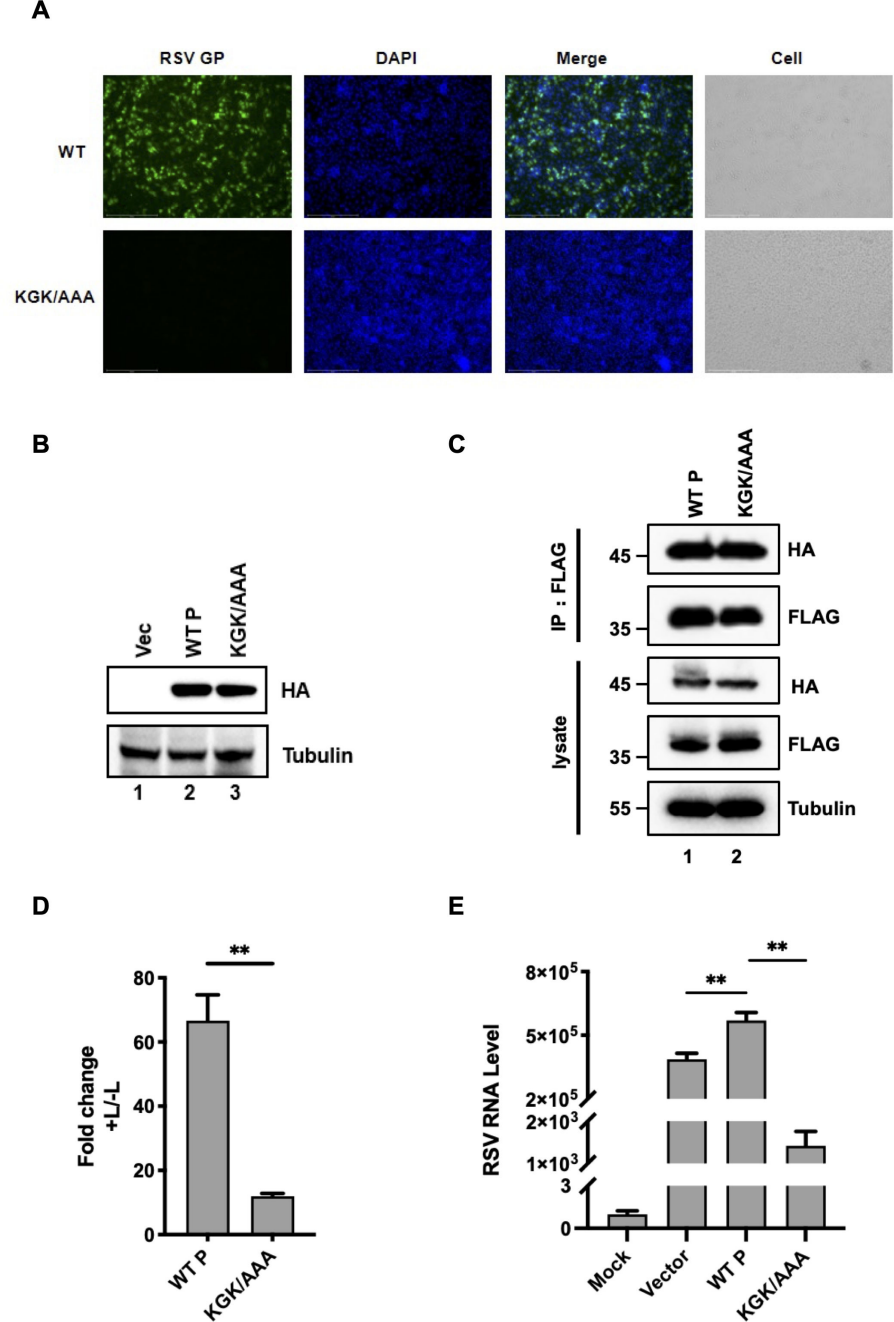
Effect of P helicase-defective KGK/AAA mutation on RSV viability. (**A**) The virus production of either WT (upper) or KGK/AAA mutant (lower) RSV A2 in Hep2 cells at 96 h was detected via immunofluorescent staining of RSV GP (green). The cell nuclei were stained with DAPI (blue). The merged image represents the digital superimposition of the green and blue signals. (**B**) 293T cells were transfected with HA-tagged WT P or the KGK/AAA mutant. At 24 h post-transfection, expression levels were assessed by Western blotting. (**C**) 293T cells were co-transfected with HA-tagged RSV N and Flag-tagged WT P or the KGK/AAA mutant. At 24 h post-transfection, N-P interactions were examined by co-immunoprecipitation and Western blotting. (**D**) BHK-21 cells were infected with vTF7-3 for 1 h and then transfected with the RSV minigenome system. Dual-luciferase activities were measured 24 h post-transfection. (**E**) H1-HeLa cells were transfected with empty vector, WT P, or the KGK/AAA mutant and were infected with RSV A2 (MOI = 0.05) 24 h later. After an additional 24 h, RSV mRNA levels were quantified by qPCR (*n* = 3; mean ± SEM; **P < 0.01, ***P < 0.001; unpaired *t*-test).

To determine whether the loss of the helicase activity of P has any consequence on the viral life cycle of RSV, we introduced the KGK/AAA mutation, which disrupts the NTPase and helicase activities, into the P coding region of the infectious clone of the recombinant full-length cDNA encoding RSV A2. Then, WT and mutant RSV cDNA were transfected into BSRT7/9 cells. Virus production was detected via immunofluorescence for the gglycoprotein (GP) in cells. Strikingly, compared to the WT virus, the KGK/AAA mutation resulted in the loss of RSV viability (Fig. 7A), suggesting that the NTPase and the RNA helicase activities of P are critical for RSV viability. In addition, we generated mutant plasmids and confirmed that the KGK mutation does not affect P protein expression (Fig. 7B) and N-P interaction (Fig. 7C). Building on these findings, we used the mutant plasmids to assess the impact of P and the KGK mutation on RSV minigenome activity and viral replication. We found that the KGK mutation markedly inhibited both minigenome replication (Fig. 7D) and infectious virus production (Fig. 7E), suggesting that the NTPase and the RNA helicase activities of P are critical for RSV replication.

## DISCUSSION

The RNA remodeling proteins, including RNA helicases and RNA chaperones, which can promote RNA molecule folding and refolding, are generally thought to play important roles in RNA metabolism (7). Currently, numerous RNA viruses have been found to encode their own RNA helicase and/or RNA chaperone (10, 11, 16, 18). In this study, we report for the first time that RSV-encoded protein P has NTPase and dsRNA-binding activities, along with RNA-helicase-like activity that efficiently unwinds RNA helix from 5′-to-3′ direction in the presence of NTP and divalent metallic ions, especially ATP and Mg^2+^. In addition, we also identified the key points P for its NTPase activities, and the NTPase activity is required for the helix-unwinding activity of RSV P. Notably, the RNA helicase activity of P is critical For the viability of RSV. Our current study has added RSV P to this growing list of virus-encoded RNA remodeling proteins.

RSV P plays critical roles in regulating RNA replication and transcription. It is intriguing to ask how the helicase activity of P functions in the life cycle of RSV. The RSV replication complex is the functional unit for viral transcription and replication, comprising the N, L, P, M2-1, and the viral RNA. During transcription and replication, the viral RNA associates with N to form N-RNA, which serves as a template for the P-bound L polymerase complex (P-L). The attachment of P-L to N-RNA occurs through the interaction between P and N proteins. Additionally, P is thought to travel along the RNP together with L, facilitating the polymerase’s translocation. In viral transcription, the interaction between P and M2-1 (P-M2-1) is essential for M2-1 to enhance L’s elongation and termination activities on the viral RNA. Although the RSV genome is a single-stranded negative-sense RNA, it has been demonstrated to generate dsRNA replication intermediates that trigger innate immunity (26, 27). P is an essential polymerase cofactor that tethers L, the RNA-dependent RNA polymerase, to the RNA replication complex (3). P also serves as a chaperone that associates with newly synthesized nascent N (28). Our dsRNA-binding assays suggest that the P may directly recognize RSV replication intermediates. Therefore, it is postulated that P can work together with L and N to mediate the unwinding of dsRNA replicative intermediates and facilitate the correct folding or refolding of the RSV mRNA and genomic RNA, thereby promoting the transcription, translation, and encapsidation of RSV.

Previous studies have shown that RSV infection activates TLR3 and RIG-I, which sense viral dsRNA or dsRNA-containing replication intermediates (26, 27). RSV P possesses canonical helicase activity and functions as a dsRNA-binding protein that can both bind and unwind dsRNA. This dual activity raises a provocative question: can a virus-encoded helicase subvert host immunity by destabilizing viral dsRNAs, thereby preventing them from being sensed by RNA sensors? Notably, RIG-I itself harbors an RNA-helicase domain, whose unwinding activity is critical for sensing viral RNA and igniting innate signaling. We therefore speculate that P antagonizes RIG-I by competing for or directly interfering with its helicase domain. Consequently, RSV P appears to be a bifunctional helicase: it simultaneously accelerates viral replication by resolving replication intermediates and dampens innate immunity by destabilizing viral dsRNA that would otherwise trigger robust antiviral responses.

RNA helicases are classified into six superfamilies, termed SF1 to SF6, according to the conserved motifs. Interestingly, RSV P does not have any traditional helicase conserved motif, but it contains the fundamental biochemical characteristics of canonical RNA helicases, including the dependence on NTP and divalent metal ions, as well as the directionality of RNA helix unwinding. Consistently, our previous study also uncovered that EBOV VP35 is an untraditional RNA helicase-like protein without any conventional helicase motif (18). Our findings suggest that some proteins associated with RNA helicase activities may not be strictly dependent on the conserved motifs in the linear amino acid sequence, but are probably functionally determined by more complex regions/sites formed by protein higher-order structures.

In summary, our work provides the first demonstration of the NTPase and helicase-like activities associated with RSV P. These findings uncover novel functions of RSV P, extend the view of RNA remodeling proteins, and shed light on the understanding of RSV replication. Furthermore, elucidating the helicase-like activity of RSV P carries profound implications for antiviral development. As a central coordinator of viral transcription and replication, P represents an attractive therapeutic target. The discovery of its enzymatic functions opens new avenues for structure-guided drug design, particularly for small molecules inhibiting NTPase/helicase activity. Furthermore, this work provides a framework for re-evaluating accessory proteins in related viruses, potentially uncovering conserved mechanisms of RNA metabolism. By bridging molecular virology and enzymology, our findings advance the fundamental understanding of RSV biology while offering tangible strategies to combat this pervasive pathogen.

## MATERIALS AND METHODS

### Plasmid and recombinant baculovirus construction

The construction of pFastBac HTB-MBP and pFastBac HTB-MBP-RSV P and pFastBac HTB-MBP-EBOV VP35 has been described previously (21). The cDNA fragments of RSV P (GenBank accession no. MK816924.1) and EBOV VP35 (GenBank accession no. AF086833.2) were cloned into the vector pFastBac HTB-MBP, where the maltose binding protein (MBP) was fused to the N-terminus. The mutations were conducted as previously described (29). The resulting plasmid was subjected to Bac-to-Bac baculovirus system to express the recombinant protein. The primers used in this study are shown in Table S1.

### Expression and purification of recombinant fusion protein

The expression and purification of recombinant MBP-P and negative control MBP from Bac-to-Bac system were conducted as previously described (9, 30). Briefly, SF9 cells were infected with the recombinant baculoviruses and harvested 72 h post-infection. Cell pellets were resuspended, lysed by sonication, and subjected to centrifugation for 15 min at 12,000 × *g* to remove debris. The protein in the supernatant was purified using amylose affinity chromatography (New England BioLabs, Ipswich, MA), according to the manufacturer’s protocol. Then the protein was concentrated using Ultra-15 filters (Millipore, Schwalbach, Germany), and the store buffer was exchanged to 50 mM 2-[4-(2-hydroxyethyl)−1-piperaziny] ethanesulfonic acid (HEPES)-KOH (pH 7.5). All proteins were quantified using the Bradford method and stored at −80℃ in aliquots. Proteins were separated by 10% SDS-PAGE and visualized with Coomassie blue.

### Preparation of oligonucleotide helix substrates

In brief, the RNA helix, DNA helix, and RNA-DNA hybrid helix were prepared by annealing two complementary nucleic acid strands. One strand was labeled at 5′ end with hexachloro-fluorescein (HEX), and the other strand was unlabeled. HEX-labeled oligonucleotide strands were purchased from TaKaRa (Dalian, China). Unlabeled DNA strands were synthesized by Invitrogen, and unlabeled RNA strands were *in vitro* transcribed using T7 RNA polymerase (Promega, Madison, WI). The transcribed RNA strands were purified by Poly-Gel RNA Extraction Kit (Omega Bio-Tek, Guangzhou, China) according to the manufacturer’s instructions. The two strands were mixed in a proper ratio and annealed through heating and gradually cooling as previously described (9, 17). The resulting duplexes were examined by 15% native-PAGE gel to ensure that all single-stranded RNA and DNA were annealed in a 1:1 ratio.

The standard RNA helix substrate (R/R*) was annealed with a 42-nt HEX-labeled single-stranded RNA1 and a 24-nt unlabeled single-stranded RNA2. The 3′-tailed and 5′-tailed RNA substrates were constructed by annealing RNA1 with a 48-nt non-labeled single-stranded RNA3 (3′-tailed) and RNA4 (5′-tailed), respectively. The blunt-ended RNA substrate was prepared by annealing RNA1 with a 42-nt non-labeled single-stranded RNA5. (D*/D) was prepared by annealing a 28-nt HEX-labeled single-stranded DNA1 with a 49-nt non-labeled single-stranded DNA2. (D/R*) was prepared by annealing RNA1 with a 30-nt non-labeled single-stranded DNA3. (R*/D) was prepared by annealing RNA1 with a 54-nt non-labeled single-stranded DNA4. All oligonucleotides used in this study are listed in Table S2.

### NTPase assay

NTPase activities were determined by measuring the inorganic phosphate released during NTP hydrolysis using a direct colorimetric assay, following the standard procedure as previously described (9). All of the results obtained from this quantitative assay represent the average of three repeated experiments.

### Gel mobility shift assay

Gel mobility shift assay was performed in 50 mM HEPES-KOH (pH 7.5), 100 mM NaCl, 2 mM MgCl_2_, 1 mM tRNA, 2 mM DTT, and 20 U RNase inhibitor (Promega) in a total volume of 10 µL reaction, with the indicated amount of protein and 0.1 pmol of dsRNA. The dsRNA was labeled with DIG-UTP (Roche) by *in vitro* transcription and derived from 200 nt EGFP. Reactions were incubated for 30 min at 25°C and terminated by the addition of 2.5 µL of 5 × sample buffer (20 mM Tris-HCl [pH 7.5], 36% glycerol, and 0.1% bromophenol blue). The nucleic acid-protein complexes were separated by electrophoresis on a 1.5% protein agarose gel and transferred to Hybond-A nylon membrane (GE Healthcare). After that, the membrane was subjected to cross-linking at 120°C and was incubated with anti-DIG-alkaline phosphatase antibody (Roche), followed by incubation with CDP-STAR (Roche) for 15 min at 37°C. The signals were then detected by X-ray film (Fujifilm, Tokyo, Japan).

### Nucleic acid helix-unwinding assay

The standard helix destabilizing assay was performed as previously described (17). Briefly, 20 pmol of recombinant protein and 0.1 pmol of HEX-labeled helix substrate were added to a mixture containing 50 mM HEPES-KOH (pH 7.5), 1 mM MgCl_2_, 100 mM NaCl, and 20 U RNase inhibitor (Promega). Reactions were incubated for 60 min at 37°C and terminated by adding 5 × loading buffer (100 mM Tris–HCl, 1% SDS, 50% glycerol, and bromophenol blue [pH 7.5]). The mixtures were then electrophoresed on 15% native-PAGE gels (15% acrylamide:bis [29:1], 89 mM Tris, 89 mM boric acid, 2 mM EDTA [pH≈ 8.3], 0.2% [wt/vol] ammonium persulfate, 0.1% [wt/vol] TEMED) in 1 × TBE buffer (89 mM Tris, 89 mM boric acid, 2 mM EDTA [pH ≈ 8.3]). The gel was run at a constant 100 V for 10–20 min to allow the HEX-labeled helix substrate to slowly enter the gel, then switched to 150 V and electrophoresis continued for approximately 40 min. The HEX-labeled helix substrate was then detected by scanning with a Typhoon 9500 imager (GE Healthcare, Piscataway, NJ).

### Construction and recovery of RSV mutant virus

To construct the plasmid for the RSV A2 strain P protein (KGK/AAA mutation), we used wild-type RSV A2 as the template. With the pCC1 vector as the backbone, we sequentially inserted a T7 promoter, three additional guanine (ggg) bases to enhance viral expression, the A2 strain genome sequence (with the KGK/AAA mutation introduced in the P gene), HDV ribozyme, and a T7 terminator to obtain the mutated viral cDNA. Meanwhile, we constructed four auxiliary plasmids encoding the N, P, L, and M2-1 proteins using the eukaryotic expression vector pCDNA3.1, resulting in pCDNA3.1-N, pCDNA3.1-P, pCDNA3.1-M2-1, and pVSV-RSV-L.

For recovery of the RSV WT and mutant (KGK/AAA) virus, BSRT7/9 cells were cultured overnight in six-well plates the day before transfection. When the cell density reached approximately 70%, the cells were transfected with the PCC1-RSV WT or KGK/AAA plasmid, along with the four helper plasmids (pCDNA3.1-N, pCDNA3.1-P, pCDNA3.1-M2-1, and pVSV-RSV-L), according to the Lipofectamine 3000 reagent manual. The transfection mixture included PCC1-RSV (WT or KGK/AAA) (1.25 µg), pCDNA3.1-N (1 µg), pCDNA3.1-P (1 µg), pCDNA3.1-M2-1 (0.25 µg), and pVSV-RSV-L (0.5 µg). After incubating the DNA-Lipofectamine-OptiMEM (Gibco) mixture at room temperature for 30 min, it was added to each well of cells. Six to 8 h post-transfection, the medium was replaced with 2% FBS DMEM. The cells were incubated at 37°C in a 5% CO₂ incubator, and cytopathic effects were observed daily. For blind passage, 4 days post-transfection, the supernatant was collected and centrifuged at 4500 × *g* for 15 min. The supernatant was then used to infect BSRT7/9 cells to increase the viral titer. The virus was subsequently transferred to Hep-2 cells for further passage and amplification.

### Immunofluorescence assay

Hep2 cells were plated in Glass Bottom Culture Dishes (NEST, 801002). After 24 h, cells were infected with WT or mutant RSV A2 for 96 h. The cells were then fixed with 4% paraformaldehyde for 45 min and permeabilized with 0.2% Triton X-100 for 20 min. After that, the cells were blocked with phosphate-buffered saline (PBS) containing 5% bovine serum albumin (BSA) for 1 h and then incubated with anti-RSV-GP antibody (1:1,000 in 5% BSA) overnight, followed by staining with FITC-labeled goat anti-mouse IgG (ABclonal) (1:1,000 in 5% BSA). Nuclei were stained with 49,6-diamidino-2-phenylindole (DAPI; Beyotime) for 5 min at 37°C in the dark. Cells were photographed under a confocal microscope (A1R; Nikon, Japan). The fluorescence intensity was analyzed using ImageJ software.

### Western blotting and antibodies

Cells were washed twice with cold PBS and lysed in lysis buffer (50 mM Tris–HCl [pH 7.4], 150 mM NaCl, 1% NP40, 0.25% deoxycholate and a protease inhibitor cocktail [MCE]). The lysates were subjected to 10% SDS-PAGE gels and transferred to polyvinylidene fluoride membranes (Bio-Rad). The blots were incubated with primary antibodies in 5% nonfat milk in TBS with 0.05% Tween 20 (TBST) overnight at 4°C. Membranes were washed in TBST and incubated in HRP-coupled secondary antibodies at room temperature for 1 h. Proteins were detected by chemiluminescence using ECL (Bio-Rad) in a Bio-Rad ChemiDoc Imager and were quantified using Image J software. The antibodies used for Western blots are as follows: the anti-HA and anti-FLAG antibodies were purchased from CST, and the anti-Tubulin and anti-GAPDH were purchased from Proteintech.

### Co-Immunoprecipitation

For the co-immunoprecipitation (co-IP) assay, cells were harvested and then lysed with IP buffer (20 mM Tris-HCl, pH 7.4, 150 mM NaCl, 10% glycerol, 2 mM EDTA, 0.5% Nonidet P-40, 0.5% Triton X-100) with 1% protease and phosphatase inhibitor (MCE). The insoluble component was removed by centrifugation at 12,000 × *g* for 10 min at 4°C, and the supernatant was collected. For each sample, 600 µL protein lysate was incubated with 1 µg antibody and 30 µL protein A/G magnetic beads (MCE) overnight at 4°C. The beads were washed five times with 1 mL of IP buffer, and then the precipitates were detected by Western blotting.

### RSV minigenome assay

BHK-21 cells were seeded in 24-well plates. After the cells have completely adhered, infect with vaccinia virus vTF7-3 and perform transfection 1 h post-infection. Cells were co-transfected with 31.25 ng pGEM4-RSV N, 62.5 ng pGEM4-RSV P, 15.625 ng pGEM4-RSV M2-1, 7.8125 ng pGEM4-RSV L, 31.25 ng pGEM4-RSV Minigenome (Firefly luciferase), and 5 ng pRL-TK (Renilla luciferase). The thymidine kinase promoter-driven Renilla luciferase plasmid (TK) was included as a transfection control. The minigenome system was driven by vTF7-3, which expresses T7 RNA polymerase. At 24 h post-transfection, cells were lysed with Passive Lysis Buffer (Promega) at room temperature for 15 min. RSV minigenome activity was quantified using the Dual-Luciferase Reporter Assay System (Promega).

## Data Availability

All data generated or analyzed during this study are included in this published article and its supplemental material.

## SUPPLEMENTAL MATERIAL

Supplemental figures jvi.00996-25-s0001.docx Figures S1 and S2.

Supplemental tables jvi.00996-25-s0002.docx Tables S1 and S2.

ASM does not own the copyrights to Supplemental Material that may be linked to, or accessed through, an article. The authors have granted ASM a non-exclusive, world-wide license to publish the Supplemental Material files. Please contact the corresponding author directly for reuse.
