# Supplementary material for: Respiratory syncytial virus phosphoprotein has NTPase and helicase-like activities
Source: J Virol. 2025 Sep 25;99(10):e00996-25. doi: 10.1128/jvi.00996-25 (PMC12548401; doi:10.1128/jvi.00996-25)
Supplement: Supplemental figures — Figures S1 and S2. [file jvi.00996-25-s0001.docx]

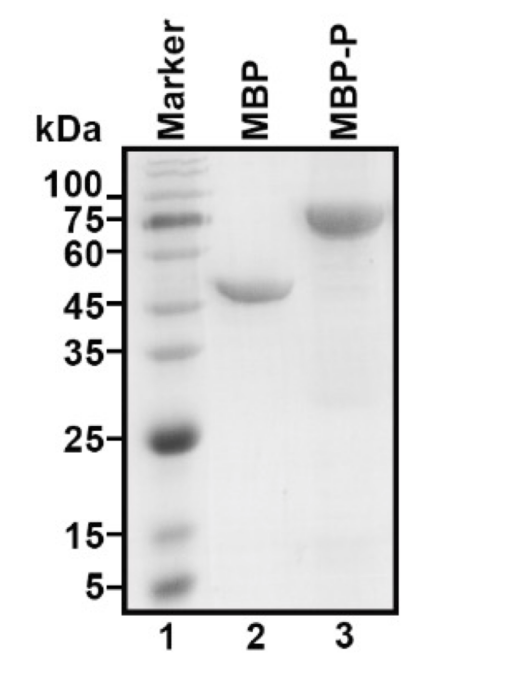


**Supplementary Figure S1. Expression of recombinant RSV P.** The MBP-fusion RSV P was expressed using a eukaryotic (baculovirus) system. The purified recombinant proteins were subjected to 10% SDS-PAGE followed by Coomassie brilliant blue R250 staining. Lane 1, protein marker; lane 2, the purified MBP alone; lane 3, the purified eukaryotically expressed MBP-P.


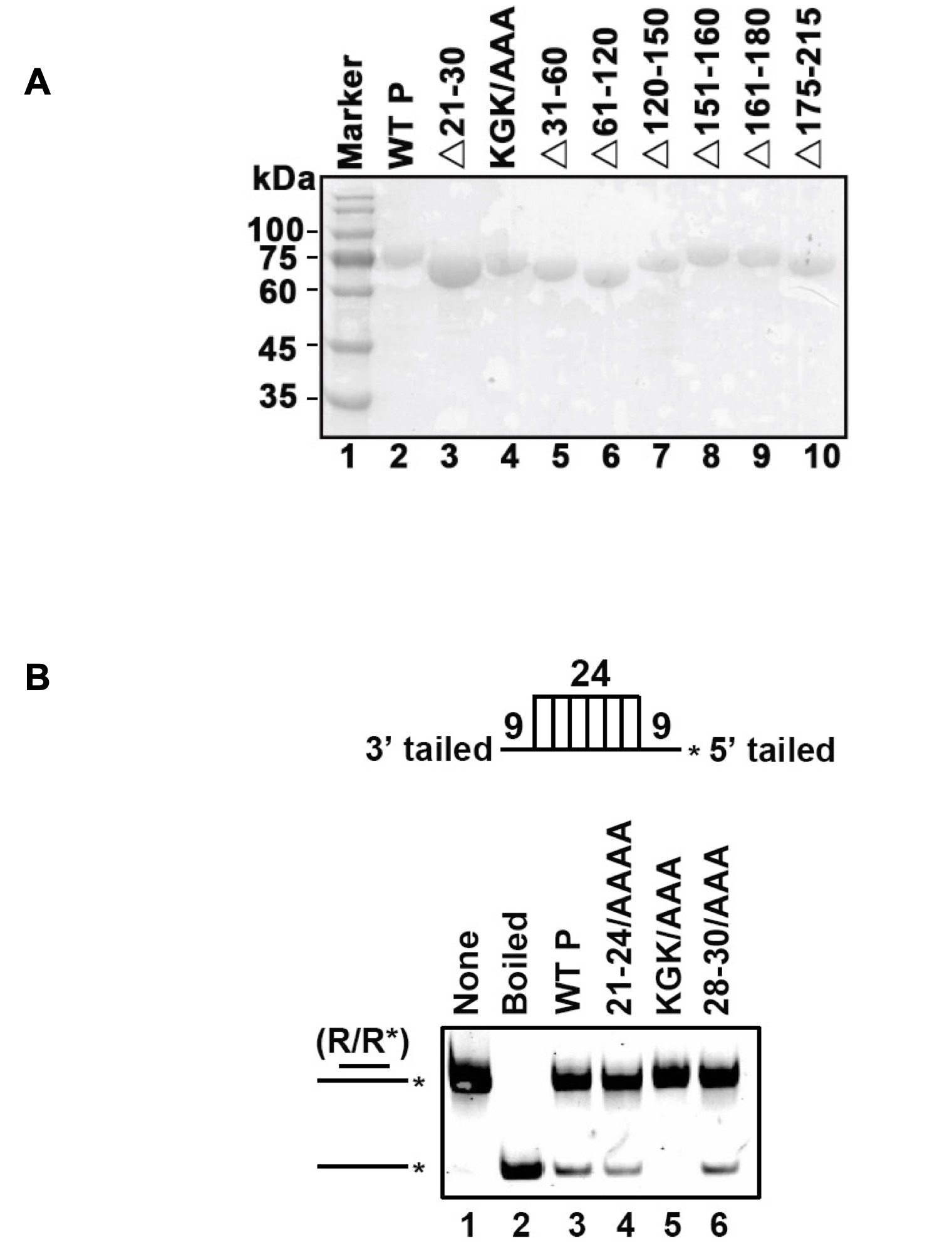


**Supplementary Figure S2. The expression and helix-unwinding activity of recombinant RSV P and its mutants. (A)** The expressed WT P, △21-30, KGK/AAA, △31-60, △61-120, △120-150, △151-160, △161-180 and △175-215 were subjected to 10% SDS-PAGE and Coomassie brilliant blue R250 staining. **(B)** The helix-unwinding activity of recombinant RSV P and its mutants. Upper panel: Schematic illustration of the standard RNA helix substrate (R/R*); asterisks indicate the HEX-labeled strands. Lower panel: The RNA helix unwinding assays were performed by incubating 0.1 pmol standard helix substrate with 20 pmol of each indicated protein.
