# Supplementary material for: Respiratory syncytial virus phosphoprotein has NTPase and helicase-like activities
Source: J Virol. 2025 Sep 25;99(10):e00996-25. doi: 10.1128/jvi.00996-25 (PMC12548401; doi:10.1128/jvi.00996-25)
Supplement: Supplemental tables — Tables S1 and S2. [file jvi.00996-25-s0002.docx]

# Supplementary Table S1

Primer used in this study

| Primer Name | Sequence |
| --- | --- |
| RSV-P-F | GCGAATTCATGGAAAAGTTTGCTCCTGAGTTCCATGGAGAAGATGC |
| RSV-P-R | GCGTCGACTCAGAAATCTTCAAGTGATAGATCATTGTC |
| △21-30-F | GGGCTACTAAATTCCCCAAAGATCCCAAGAAAAAAGATAG |
| △21-30-R | GGGATCTTTGGGGAATTTAGTAGCCCTGTTGTTTGCATC |
| KGK/AAA-F | TAGAATCAATAGCGGCCGCATTCACATCACCCAAAGATCCCAAGAAAAAA |
| KGK/AAA-R | TGATGTGAATGCGGCCGCTATTGATTCTAGGAATTTAGTAGCCCTGTT |
| △31-60-F | GGGCAAATTCACATCAACTATTATCAACCCAACAAATGAGACAG |
| △31-60-R | GGGTTGATAATAGTTGATGTGAATTTGCCCTTTATTGATTCTAGG |
| △61-120-F | GCCCTATAACATCAAATTCAGAAGAAATAAATGATCAGACAAACG |
| △61-120-R | GATCATTTATTTCTTCTGAATTTGATGTTATAGGGCTTTCTTTGG |
| △120-150-F | GAAGAAGAATCCAGCTATTCAACATTAGTAGTGGCAAGTG |
| △120-150-R | CCTGCACTTGCCACTACTAATGTTGAATAGCTGGATTCTTCTTCATTG |
| △151-160-F | CTAGGAATGCTTCACTCTGCTCGGGATGGTATAAGAGATGCC |
| △151-160-R | CCATCCCGAGCAGAGTGAAGCATTCCTAGTATTTCACTTAATTTTTCATC |
| △161-180-F | GCAGGACCTACAATCAGAACTGAAGCATTAATGACCAATGACAG |
| △161-180-R | GCTTCAGTTCTGATTGTAGGTCCTGCACTTGCCACTAC |
| △175-215-F | GGTTGGTTTAAGACTCAATCCAACATCAGAGAAATTGAACAACC |
| △175-215-R | GTTGGATTGAGTCTTAAACCAACCATGGCATCTCTTATACCATCCCG |

# Supplementary Table S2

The sequences of oligonucleotides used for RNA helix, DNA helix and hybrid helix.

| Oligonucleotide | Sequence（5’-3’） |
| --- | --- |
| RNA1* | **CAUUAUCGGAUAGUGGAACCUAGCUUCGACUAUCGGAUAAUC** |
| RNA2 | ATAGTCGAAGCTAGGTTCCACTAT |
| RNA3 | GAUUAUCCGAUAGUCGAAGCUAGGUUCCACUAUCCGAUAAUGAAAUAA |
| RNA4 | AAUAAAGAUUAUCCGAUAGUCGAAGCUAGGUUCCACUAUCCGAUAAUG |
| RNA5 | GAUUAUCCGAUAGUCGAAGCUAGGUUCCACUAUCCGAUAAUG |
| DNA1* | **CACCACAACCACCACCACCACACCATGG** |
| DNA2 | TGTAGTGCTGCCATGGTGTGGTGGTGGTGGTTGTGGTGGAGCTACGAAC |
| DNA3 | CCGATAGTCGAAGCTAGGTTCCACTATCCG |
| DNA4 | AATAAAGATTATCCGATAGTCGAAGCTAGGTTCCACTATCCGATAATGAAATAA |

*HEX-labeled strands are in boldface.
